# Iterative Amplitude Equalization for Frequency Estimation (IAE-DFT)

**DOI:** 10.3390/s25237344

**Published:** 2025-12-02

**Authors:** Elena Serea, Codrin Donciu, Marinel Costel Temneanu

**Affiliations:** Faculty of Electrical Engineering, “Gheorghe Asachi” Technical University of Iași, 700050 Iași, Romania

**Keywords:** frequency estimation, spectral leakage, signal to noise ratio, embedded measurement systems, iterative amplitude equalization

## Abstract

The accurate frequency estimation of sinusoidal signals remains a key requirement in precision instrumentation and signal analysis, particularly in applications where noise and spectral leakage affect the measurement accuracy. This paper introduces a new frequency-domain technique, called IAE-DFT (Iterative Amplitude Equalization in the Frequency Domain), which estimates the true frequency of a sinusoidal component by iteratively adjusting two dominant spectral points until their amplitudes become balanced. Both spectral components are shifted together in the same direction according to amplitude dominance, and the step size is halved each time the amplitude relationship reverses, ensuring smooth and deterministic convergence. Experimental results demonstrate that IAE-DFT achieves superior performance at 0 dB SNR, outperforming the state-of-the-art methods, while maintaining comparable accuracy at 20 dB and 40 dB. Its precision and robustness make it a promising candidate for frequency-output biosensors and resonant sensing applications, where accurate tracking of small frequency shifts is critical. Future work will focus on optimizing the iteration control strategy, particularly the selection of the initial step size and the adaptive adjustment rate, to further enhance convergence speed and accuracy.

## 1. Introduction

Accurate frequency estimation of a dominant sinusoidal component remains a fundamental and widely studied problem in digital signal processing, instrumentation and metrology. Practical measurement systems often rely on the extraction and tracking of a single spectral component, for example, in frequency metering and synchronization in electrical power grids [1], controlled single-tone excitation in resonance monitoring and structural testing [2,3], Doppler sensing in active radar and sonar systems where a single shifted tone is analyzed [4,5], carrier tracking in communication receivers [6], carrier beat-frequency locking in coherent inter-satellite laser communication systems [7] and quantum frequency standards where the precession frequency of a single atomic transition defines the measured signal [8]. In all these domains, small deviations in frequency carry essential physical or diagnostic information and must be estimated with high precision.

Although estimating the frequency of a single sinusoidal tone may appear straightforward, the problem becomes substantially more complex when considered under real-world conditions. When signals are discretely sampled over finite time intervals, the resulting spectrum is affected by quantization in the frequency domain and by leakage of energy from the main spectral lobe into adjacent bins. These effects, known, respectively, as the picket-fence effect and spectral leakage [9], can significantly bias amplitude, phase and frequency estimates. In practice, the spectrum of a discrete-time sinusoid is typically obtained through the Fast Fourier Transform (FFT), which efficiently computes the Discrete Fourier Transform (DFT) of the sampled signal. However, the resolution of the FFT is limited by the ratio between the sampling frequency and the number of samples, and the frequency grid is discrete [10]. If the true signal frequency lies between two FFT bins, the spectral maximum appears shifted and broadened. This leads to systematic errors that grow with smaller data records or non-coherent sampling conditions.

To mitigate leakage, window functions such as Hann, Hamming, or Blackman are commonly applied prior to the FFT [11]. These windows smooth the signal edges and suppress sidelobes, but they also broaden the main lobe and reduce amplitude accuracy [12]. Another common enhancement is zero-padding, which extends the time-domain signal by adding zeros before performing the FFT. Although zero-padding does not increase the true frequency resolution, it refines the spectral sampling grid, providing a smoother and more continuous representation of the signal’s spectrum [13]. This technique is particularly helpful for identifying the approximate peak location and for enabling sub-bin interpolation between FFT lines.

Over the past four decades, numerous methods have been proposed to extract accurate frequency estimates from FFT data while compensating for the limitations of discrete sampling and finite observation windows. Among the most influential are the Interpolated DFT (IpDFT) and its numerous descendants, which have evolved into fractional, hybrid and generalized forms. Each method aims to achieve higher precision and robustness without sacrificing computational efficiency, which remains essential for real-time measurement systems and embedded devices. The origin of frequency-domain interpolation methods can be traced back to the seminal work in [14], proposing a high-accuracy spectrum analysis technique using analytical leakage compensation. The, known today as the Interpolated DFT (IpDFT), established a mathematical relationship between the amplitudes of the dominant FFT line and its neighboring lines. By exploiting this relationship, the IpDFT was able to analytically compensate for leakage and produce unbiased estimates of amplitude, phase and frequency. This classical method proved to be both conceptually clear and computationally efficient. However, its accuracy depends strongly on the position of the true frequency relative to the FFT bins. When the signal frequency coincides with a bin center, the neighboring spectral lines have very small amplitudes, and the interpolation process becomes highly sensitive to noise and rounding errors. Furthermore, the method assumes an ideal rectangular window, which can introduce additional bias when other window types are used [15]. Despite these limitations, the IpDFT became a cornerstone for all subsequent frequency estimators based on the FFT. Recent developments continue to refine leakage-compensation strategies, including adaptive two-point estimators and generalized IpDFT extensions that improve robustness under low-SNR and off-bin conditions [16,17].

A notable extension was proposed by [18], who combined zero-padding with fractional-frequency evaluation in a hybrid FFT–DTFT interpolation approach. Their method first applies zero-padding to increase spectral density, producing a smoother FFT spectrum that approximates the continuous DTFT. Next, the algorithm evaluates several points around the main spectral lobe at fractional frequency offsets and applies an interpolation formula to estimate the true peak position. The advantage of this approach lies in its ability to exploit both the computational efficiency of the FFT and the precision of fractional DTFT evaluation. It achieves sub-bin accuracy without requiring excessively long data records or iterative optimization. However, the use of zero-padding and multiple DTFT computations increases the total processing time compared to classical IpDFT. Still, Fan’s method represents an important step toward practical high-resolution spectral estimation in applications such as acoustic sensing, communications and vibration diagnostics.

To address the inherent sensitivity of integer-bin interpolation, researchers introduced fractional-frequency evaluation techniques. A major step forward was made by [19] with the Fractional Interpolated DFT (FracIpDFT). This method computes the spectral components not only at integer FFT bins but also at fractional positions, typically at half-bin intervals. By doing so, it effectively bridges the gaps between bins and provides a smoother estimation of the true peak position. The FracIpDFT employs non-integer Goertzel filters to evaluate the Discrete-Time Fourier Transform directly at the desired fractional frequencies, avoiding the need for additional FFT computations. The algorithm demonstrated strong performance at low and moderate signal-to-noise ratios, where conventional IpDFT methods tend to degrade.

Building on these earlier concepts, Ref. [20] introduced the Generalized Fractional Interpolated DFT (GFIpDFT), which unifies and extends previous fractional methods. Their formulation allows an arbitrary fractional spacing between DTFT samples, rather than being limited to half-bin intervals. The GFIpDFT evaluates three symmetrically spaced frequency samples around the dominant FFT line and derives a closed-form correction term for the frequency offset. This generalization makes it possible to fine-tune the fractional spacing to the characteristics of the window function or the expected SNR, offering additional flexibility. The GFIpDFT achieves nearly optimal accuracy for low and moderate SNR levels, with errors approaching the theoretical Cramér–Rao lower bound. Another advantage is that it operates non-iteratively, producing fast results suitable for real-time applications. Nevertheless, GFIpDFT exhibits a minor but systematic degradation in accuracy at very high SNR (typically above 40 dB). This effect stems from approximations in its analytical model, which rely on a real-valued correction term and neglect certain complex interactions within the main spectral lobe. The authors themselves observed that deterministic bias becomes significant when the spectral peak is extremely narrow and symmetric, a condition typical of coherent sampling and high-quality measurements.

Given this context, the method presented in this paper introduces an iterative frequency estimation technique based on a successive amplitude-equalization principle. The method builds upon the observation that, the true frequency lies at the midpoint between two symmetric frequency samples whose spectral amplitudes are equal. In other words, when two spectral evaluations located symmetrically around the dominant FFT bin exhibit identical magnitude, their average frequency corresponds to the actual tone frequency.

## 2. Materials and Methods

When a sinusoidal signal is sampled over a non-integer number of periods, its energy is no longer concentrated in a single FFT bin. Instead, a portion of the spectral energy spreads into adjacent bins—a phenomenon known as spectral leakage. This effect is inherent to finite-length sampling and depends on the signal’s frequency relative to the FFT grid and on the type of window function applied. Spectral leakage alters the shape of the magnitude spectrum, broadening the main lobe and introducing slight asymmetries that complicate the accurate determination of the true signal frequency.

**Figure 1:**
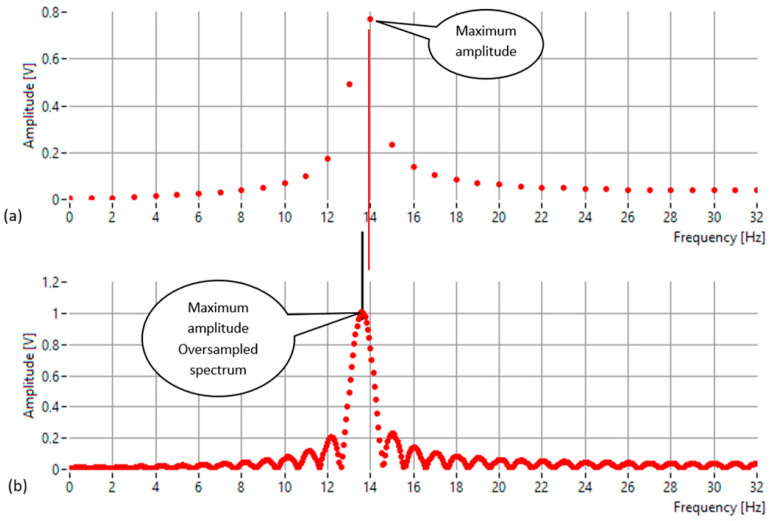
The frequency-domain representation of a 13.6 Hz sinusoidal signal (amplitude = 1 V, 64 samples at 64 Hz): (**a**) Standard FFT spectrum showing the peak at 14 Hz—the nearest discrete bin—illustrating the quantization error introduced by the finite frequency resolution of 1 Hz; (**b**) Oversampled (interpolated) spectrum obtained via zero-padding, providing a finer frequency grid that accurately reveals the true spectral maximum at 13.6 Hz.

Figure 1 illustrates this behavior for a sinusoidal signal with an amplitude of 1 V, sampled over 64 points at a rate of 64 samples per second. The true frequency of the signal is 13.6 Hz, while the FFT frequency resolution is 1 Hz. As shown in Figure 1a, the peak magnitude occurs at 14 Hz, which corresponds to the nearest FFT bin but not to the actual frequency. The difference arises because the discrete frequency grid of the FFT can represent only integer multiples of the frequency resolution. As a result, the spectral maximum is shifted toward the closest bin, leading to a small but systematic estimation error.

To obtain a more accurate estimate of the true frequency, frequency-domain oversampling or interpolation can be used. By increasing the number of frequency points—either through zero-padding the time-domain signal or by evaluating the Discrete-Time Fourier Transform (DTFT) at fractional frequency intervals—the main spectral lobe can be sampled with much finer granularity. The oversampled spectrum (Figure 1b) provides a continuous representation of the signal’s magnitude response, clearly revealing the true location of the amplitude maximum. This oversampling forms the foundation for high-precision frequency estimation methods.

Nevertheless, conventional interpolation techniques may still produce small biases, particularly when the signal frequency lies close to the midpoint between two FFT bins or when the windowed spectrum exhibits near-perfect symmetry. In such cases, simple parabolic or ratio-based interpolators can become unstable or less accurate. To address this limitation, the present study introduces a new approach focused exclusively on frequency estimation through a successive amplitude-equalization process.

The proposed method operates directly on the magnitude spectrum and relies on the observation that the true frequency of a sinusoidal component corresponds to the point where the spectrum is locally symmetric. Practically, this symmetry is achieved when two frequency samples—located symmetrically around the main spectral peak—exhibit equal amplitude. The algorithm therefore iteratively adjusts the positions of these two frequency points, one on each side of the main lobe, until their amplitudes become equal within a predefined tolerance. Once this condition is reached, the estimated frequency is defined as the average of the two frequencies with equal amplitude. This approach eliminates the need for phase analysis or analytical correction formulas. It focuses entirely on the amplitude domain, using the natural symmetry of the spectral envelope as the indicator of the true frequency.

The rationale behind the equal-amplitude criterion is rooted in the local symmetry of the DTFT magnitude response of a single-tone sinusoid. For a deterministic sinusoid in noise-free conditions, the DTFT magnitude is an even function around the true frequency, meaning that any two frequencies equidistant from the peak have identical magnitude. Consequently, enforcing the equal-amplitude condition ensures that the midpoint between the two evaluation frequencies converges to the true tone frequency. Since the proposed method enforces this symmetry iteratively, the resulting estimator is asymptotically unbiased in the absence of noise. The presence of noise perturbs the symmetry but does not alter its expectation, which explains the behavior observed in the Monte Carlo simulations.

Unlike GFIpDFT and related three-point estimators, which compute a closed-form correction term using three fixed spectral samples, the proposed method performs no analytical interpolation. Instead, it iteratively shifts two spectral evaluations while monitoring the amplitude-dominance sign and convergence is reached when the two amplitudes become equal. This symmetry-based search mechanism represents a mathematically distinct approach compared to fractional interpolators.

The algorithm begins by computing the FFT of the sampled signal and identifying the two bins with the largest amplitudes, denoted M_1_ and M_2_. These bins are typically adjacent and located on either side of the main spectral lobe. Their amplitudes represent the initial reference values for the iterative process. At each iteration, the amplitudes of M_1_ and M_2_ are recalculated using single-point DFT evaluations, which allow amplitude computation at arbitrary (possibly fractional) frequency positions between FFT bins. The comparison between these amplitudes determines the direction of movement for both indices:

- If M_1_ (the lower-frequency bin) has the higher amplitude, it means the true frequency is located toward smaller frequencies. In this case, both indices are shifted one step to the left (toward lower frequency).
- Conversely, if M_2_ (the higher-frequency bin) has the higher amplitude, the true frequency lies toward larger frequencies, and both indices are shifted one step to the right.

Thus, during each iteration, both points move together in the same direction, maintaining their relative spacing while scanning the spectral region around the peak. After each movement, the amplitudes are recalculated, and the algorithm checks whether the side with the larger amplitude has changed. When the amplitude dominance switches from one point to the other—meaning that the equal-amplitude region has been crossed—the algorithm reduces the step size by half and reverses the direction of movement.

This adaptive adjustment of the step size allows the algorithm to progressively “zoom in” on the point of balance where the amplitudes of M_1_ and M_2_ become equal. The process continues iteratively until a maximum number of iterations is performed. Once the amplitudes are equal (within tolerance), the estimated frequency is obtained as the average of the current frequency indices of M_1_ and M_2_. This midpoint corresponds to the frequency where the spectral lobe is perfectly balanced in amplitude, representing the most accurate estimate of the true sinusoidal frequency.

**Figure 2:**
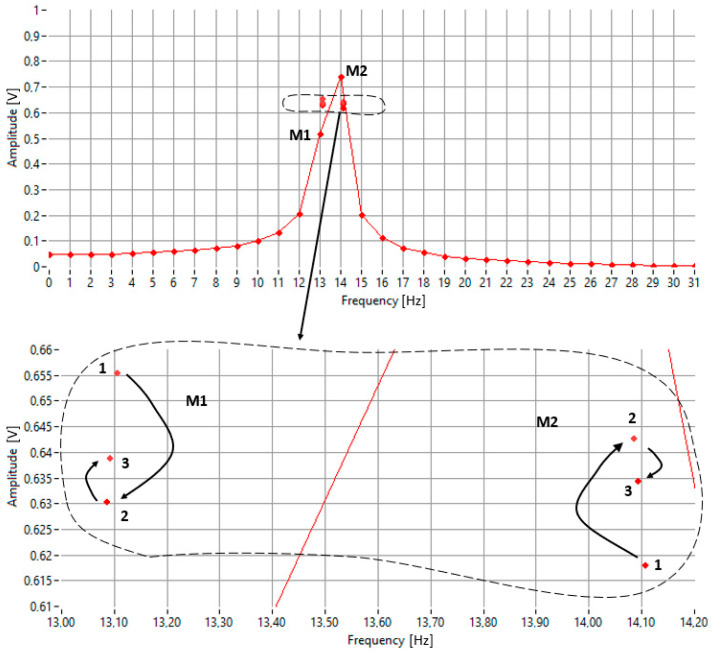
An illustration of the iterative frequency-search process used by the proposed algorithm.

In the example shown in Figure 2, the goal is to decrease the amplitude of M_2_ and increase that of M_1_ until they become equal. This requires shifting the pair to the right. The zoomed-in detail of the figure illustrates three steps of successive approximation:

- Step 1: M_1_ < M_2_, rightward shift, new pair labeled (1);
- Step 2: M_1_ > M_2_, leftward shift, new pair labeled (2);
- Step 3: M_1_ < M_2_, rightward shift, new pair labeled (3).

**Figure 3:**
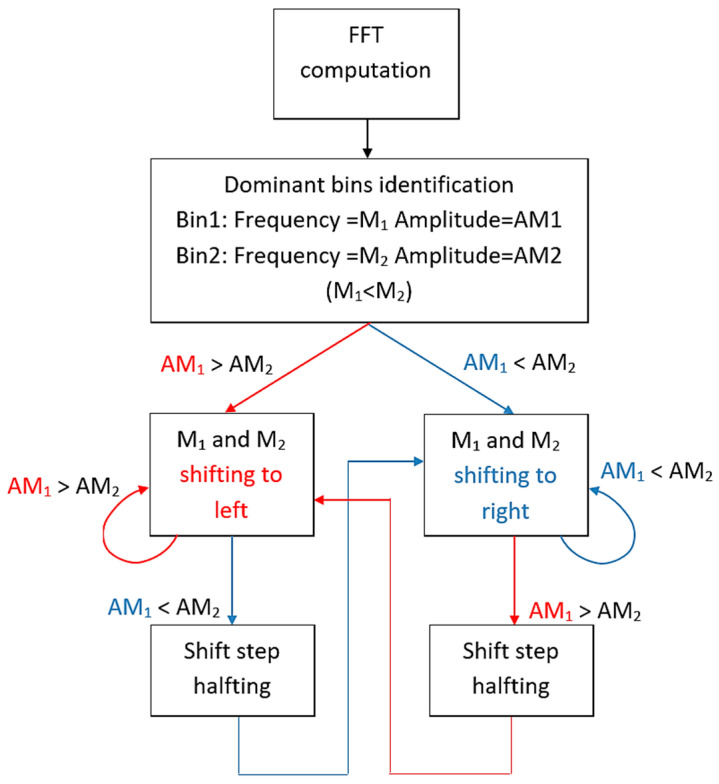
A flow diagram of the IAE_DFT algorithm.

The complete procedure of the proposed IAE-DFT algorithm is summarized in Appendix A, where the pseudocode provides a clear step-by-step description of the iterative equalization process, including the initialization, update rule, step-halving logic and termination conditions. It outlines the main computational steps used for iterative amplitude equalization and frequency estimation. The flow diagram is shown in Figure 3.

Step 1—FFT computation:

The algorithm starts by applying the Fast Fourier Transform to the sampled signal in order to obtain its discrete frequency spectrum. This provides the set of amplitude values used to locate the region containing the true frequency component.

Step 2—Identification of dominant bins:

From the FFT spectrum, the two adjacent frequency components with the largest amplitudes are selected. These correspond to the left and right sides of the main spectral lobe and serve as the initial frequency positions, denoted M_1_ and M_2_.

Step 3—Initialization:

An initial frequency step is defined as 0.4 × Δf, where Δf is the FFT frequency resolution. The initial amplitudes of M_1_ and M_2_ are calculated, and their relative dominance is recorded to determine the direction of movement in the first iteration.

Step 4—Iterative adjustment:

At each iteration, both frequency points are moved in the same direction. If the lower-frequency component (M_1_) has a higher amplitude, both are shifted toward smaller frequencies; if the higher-frequency component (M_2_) dominates, both move toward larger frequencies. After each shift, the amplitudes are recomputed using single-point DFT evaluations to obtain accurate values between FFT bins. Whenever the amplitude dominance changes from one side to the other, the step size is halved to refine the search region. This process continues until the maximum number of iterations is reached.

Step 5—Frequency estimation:

After the iterative process completes, the estimated frequency f_est_ is obtained as the average of the final values of M_1_ and M_2_. This midpoint represents the frequency where the spectral envelope is locally balanced and corresponds to the true sinusoidal frequency.

The effectiveness of the initial step size depends primarily on the true frequency position relative to the FFT bins, where δ = f_true_ − kΔf denotes the frequency offset from the nearest FFT line. When the sinusoid lies near the midpoint between two FFT lines (|δ| ≈ Δf/2), the main-lobe asymmetry is large and the amplitudes of M_1_ and M_2_ differ significantly, making larger steps efficient and stable. For intermediate offsets (|δ| ≈ Δf/4), step sizes in the range 0.3–0.5 Δf provide consistent convergence. In contrast, when the true frequency lies very close to a bin center (|δ| < 0.1 Δf), the spectrum becomes nearly symmetric and large steps may overshoot the equal-amplitude point, increasing the number of halving cycles. The default value of 0.4 Δf therefore represents a robust compromise that performs well for all relative frequency positions.

## 3. Results

To assess the performance of the proposed approach, a comparative analysis was carried out encompassing the IpDFT, FracIpDFT, Fan and GFIpDFT methods, with the theoretical Cramér–Rao Lower Bound (CRLB) employed as the optimal reference limit:

(1) $$CRLB=\frac{f_{s}}{2\pi }\sqrt{\frac{12}{N\cdot (N^{2}-1)\cdot SNR}}$$

where *f_s_* is the sampling frequency, *N* is the points number, and the *SNR* expresses the signal to noise ratio of the signal, defined as ratio between the signal power of a pure sinusoid *A*^2^/2 and the noise power *σ*^2^:

(2) $$SNR=\frac{A^{2}}{2\sigma^{2}}$$

A sinusoidal signal sampled at a frequency of *f_s_* = 512 Hz was used, with an amplitude of 2^1/2^ and a total of *N* = 512 samples. The signal frequency was swept from 126.5 Hz to 127.5 Hz, corresponding to a span equal to the frequency resolution Δf, with an incremental step of 0.005 Hz. To ensure statistically reliable RMSE values at each frequency point, 100,000 Monte Carlo repetitions were used, matching the evaluation protocol of the reference methods for fair comparison. Comparative evaluations were conducted at three SNR levels—0 dB, 20 dB and 40 dB—using additive Gaussian noise with a standard deviation of *σ*.

### 3.1. SNR = 0 dB

**Figure 4:**
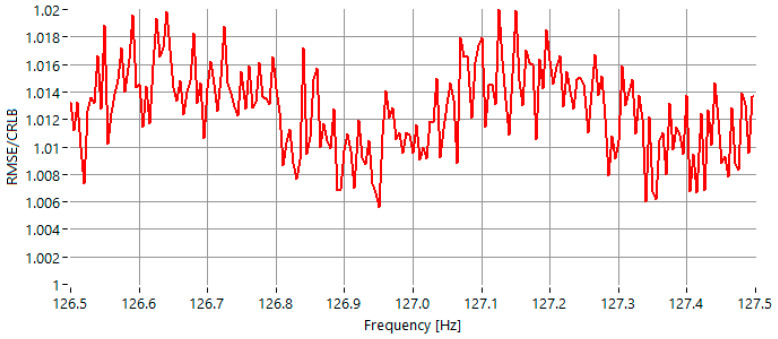
RMSE of the estimated frequency, normalized to the CRLB for sinewaves with frequency in the range 126.5 Hz–127.5 Hz, for *SNR* = 0 dB.

In the first test scenario, the signal-to-noise ratio (SNR) was set to 0 dB, and the frequency of the sinusoidal signal was varied within the range of 126.5 Hz to 127.5 Hz. As illustrated in Figure 4, the maximum observed value of the RMSE-to-CRLB ratio is approximately 1.020.

At a signal-to-noise ratio of 0 dB, the proposed IAE-DFT method demonstrates the best overall performance among all evaluated algorithms. As shown in Table 1, the maximum RMSE/CRLB ratio for IAE-DFT is 1.020, slightly lower than the best reference method, GFIpDFT, which reaches 1.025. This indicates that the iterative amplitude equalization approach maintains high accuracy even under extremely noisy conditions. Unlike classical interpolation techniques, which rely on analytical corrections that become unstable in low-SNR scenarios, IAE-DFT iteratively balances amplitude symmetry, allowing it to reject noise effects more effectively. Overall, the IAE-DFT outperforms all other methods (IpDFT, FracIpDFT, Fan and GFIpDFT) and closely approaches the theoretical Cramér–Rao lower bound, confirming its suitability for highly noisy environments.

### 3.2. SNR = 20 dB

**Figure 5:**
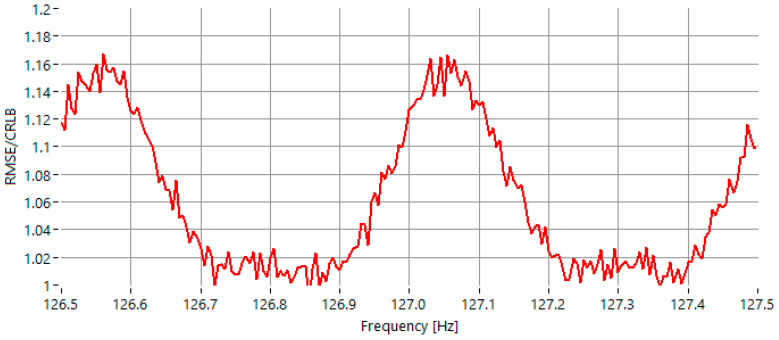
RMSE of the estimated frequency, normalized to the CRLB for sinewaves with frequency in the range 126.5 Hz–127.5 Hz, for *SNR* = 20 dB.

The second test case scenario was conducted under 20 dB and the frequency of the sinusoidal signal was varied within the same range. As shown in Figure 5, the maximum value of the RMSE/CRLB ratio is approximately 1.16.

For an SNR of 20 dB, the IAE-DFT method achieves comparable performance to the GFIpDFT, with both methods exhibiting a maximum RMSE/CRLB ratio of approximately 1.15. This result confirms that the iterative equalization process continues to produce accurate and stable estimates even when the signal quality improves, maintaining precision equivalent to state-of-the-art fractional interpolation techniques. At this moderate SNR, both IAE-DFT and GFIpDFT outperform the earlier IpDFT and Fan methods, which exhibit larger deviations near spectral bin boundaries.

### 3.3. SNR = 40 dB

**Figure 6:**
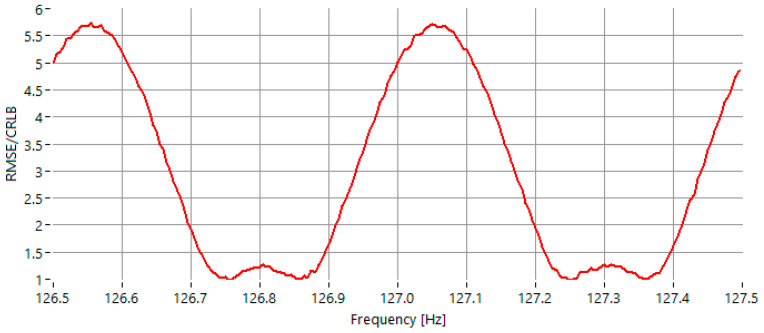
RMSE of the estimated frequency, normalized to the CRLB for sinewaves with frequency in the range 126.5 Hz–127.5 Hz, for *SNR* = 40 dB.

In the last experimental scenario, the signal-to-noise ratio was fixed at 40 dB, while the frequency of the sinusoidal signal was similarly varied within the range of 126.5 Hz to 127.5 Hz. As depicted in Figure 6, the maximum value of the RMSE-to-CRLB ratio was approximately 5.7.

At a high signal-to-noise ratio of 40 dB, the IAE-DFT method shows comparable accuracy to GFIpDFT, with both methods achieving RMSE/CRLB ratios close to their theoretical limits. As Table 1 indicates, the IAE-DFT yields a maximum ratio of 5.7, similar to GFIpDFT (5.35), with the minimum ratio reaching unity, indicating convergence to the ideal limit for certain frequencies.

**Table 1:** A comparative summary between the proposed method (IAE-DFT) and the references IpDFT, FracIpDTF, Fan and GFIpDTF for all the tested cases.

Method	Author, Year	SNR	RMSE/CRLB Max	RMSE/CRLB Min	Worst RMSE Value
IpDFT	Renders, 1984	0 dB	2.20	1.15	when the frequency of the waveform coincides with a FFT point
FracIpDTF	Rodrigues, 2022		1.40	1.15	near kΔf ± Δf/4 (where k is an integer)
Fan	Fan, 2020		1.20	1.15	near kΔf ± Δf/4 (where k is an integer)
GFIpDTF	Janeiro, 2025		1.025	1.000	near kΔf ± Δf/4 (where k is an integer)
IAE-DFT	Proposed method		1.020	1.006	-
IpDFT	Renders, 1984	20 dB	2.20	1.00	when the frequency of the waveform coincides with a FFT point
FracIpDTF	Rodrigues, 2022		1.19	1.00	near kΔf ± Δf/4 (where k is an integer)
Fan	Fan, 2020		1.25	1.15	near kΔf ± Δf/4 (where k is an integer)
GFIpDTF	Janeiro, 2025		1.15	1.05	near kΔf ± Δf/4 (where k is an integer)
IAE-DFT	Proposed method		1.16	1.01	near kΔf and kΔf ± Δf/2 (where k is an integer)
IpDFT	Renders, 1984	40 dB	2.00	1.00	when the frequency of the waveform coincides with a FFT point
FracIpDTF	Rodrigues, 2022		1.50	1.00	near kΔf ± Δf/4 (where k is an integer)
Fan	Fan, 2020		2.10	1.00	near kΔf ± Δf/4 (where k is an integer)
GFIpDTF	Janeiro, 2025		5.35	1.40	near kΔf ± Δf/4 (where k is an integer)
IAE-DFT	Proposed method		5.7	1	near kΔf and kΔf ± Δf/2 (where k is an integer)

Because all evaluated estimators exhibit negligible bias under the tested conditions, the RMSE reduces to the standard deviation of the estimator.

**Figure 7:**
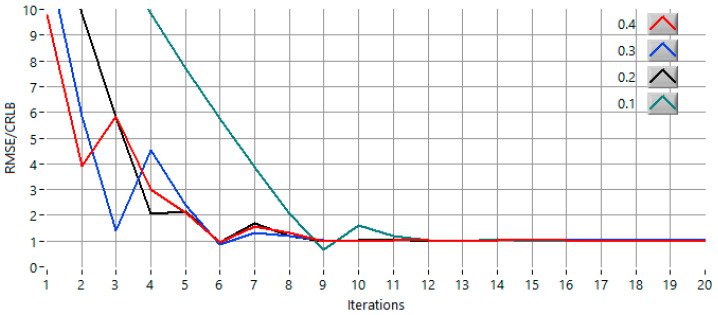
The convergence behavior of the proposed IAE-DFT method for different initial step sizes (0.4·Δf, 0.3·Δf, 0.2·Δf and 0.1·Δf) in the most stringent case test scenario (*SNR* = 0 dB, f = 127.15 Hz).

For the method to remain convergent, the initial step must be smaller than half of the FFT frequency spacing (Δf/2). If the step is equal to or larger than 0.5·Δf, the two evaluation points may overshoot the symmetry region in a single iteration, which leads to divergence or oscillatory behavior. Within the valid convergence interval, however, the convergence speed increases as the initial step approaches this upper limit. Figure 7 shows the evolution of the RMSE/CRLB ratio across iterations for different initial step sizes (0.4·Δf, 0.3·Δf, 0.2·Δf and 0.1·Δf) in the most demanding scenario (0 dB *SNR*, f = 127.15 Hz). All configurations converge toward the theoretical limit, although the number of iterations varies with the step choice, confirming that the proposed IAE-DFT algorithm remains stable and convergent across a broad range of step-size settings, even under severe noise conditions.

**Figure 8:**
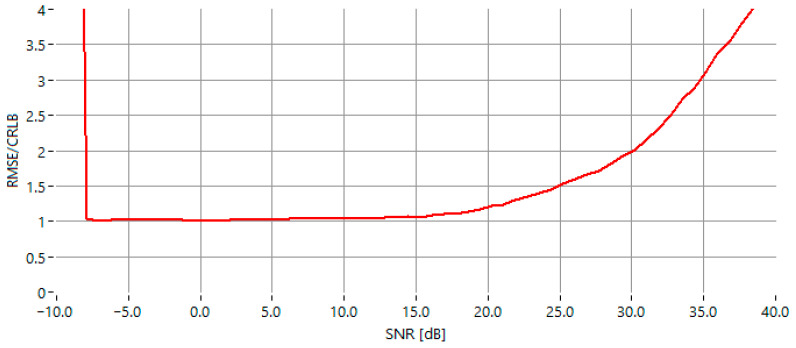
The RMSE/CRLB ratio as a function of SNR for the proposed IAE-DFT estimator.

The evolution of the RMSE/CRLB ratio as a function of SNR (Figure 8) shows a clear transition from a noise-dominated region to a range where the estimator operates close to its optimal accuracy. For lower SNR values (approximately below −7.5 dB), the ratio is noticeably higher, reflecting the strong influence of noise on the amplitude symmetry used by the algorithm. As the SNR increases from about −7.5 dB to 15 dB, the curve stabilizes into a broad plateau where the RMSE remains close to the theoretical bound, indicating robust and consistent performance. At higher SNR levels, the ratio begins to rise again. Overall, the plot demonstrates a wide mid-SNR region in which the estimator provides near-optimal precision.

## 4. Discussion

The results obtained for the three noise levels confirm that the proposed IAE-DFT algorithm maintains consistent estimation accuracy across all tested conditions.

At low SNR (0 dB), it outperforms all compared techniques, including GFIpDFT, which is known for its excellent noise resilience. This demonstrates the strength of the amplitude-equalization approach, which does not rely on explicit analytical correction terms or phase information. At medium and high SNR values (20 dB and 40 dB), the IAE-DFT maintains accuracy comparable to GFIpDFT, confirming that its iterative mechanism remains effective even when spectral leakage or bin alignment effects dominate the error sources.

Another important advantage of the IAE-DFT is its numerical stability across all tested frequencies, including those near the FFT bin centers, where ratio-based estimators typically degrade. The iterative equalization mechanism automatically adapts to the local shape of the spectral lobe, converging smoothly regardless of the initial position of the peak. This characteristic makes the algorithm particularly well suited for embedded measurement systems and biosensing applications, where deterministic convergence and low resource usage are essential.

Despite these favorable results, several aspects of the algorithm can be further optimized. In particular, the selection of the initial step size (expressed as a fraction of the FFT frequency resolution Δf) and the step-halving rate strongly influence both the convergence speed and the final estimation accuracy. A systematic evaluation of these parameters across realistic sensor signal conditions would provide insight into the method’s robustness and facilitate its practical deployment. Furthermore, quantifying the trade-off between computational complexity could support the design of energy-efficient sensor systems with predictable performance.

It is important to note that the proposed method assumes a predominantly single-tone spectrum. When periodic signals contain significant higher harmonics, the symmetry of the main spectral lobe may be locally distorted by the overlapping contribution of nearby harmonic components. In such cases, the iterative amplitude-equalization process may converge more slowly or exhibit small biases, particularly when a harmonic partially overlaps with the frequency interval scanned by the algorithm. These effects are typical for amplitude-based estimators and can be mitigated through harmonic suppression techniques, such as narrowband filtering, window selection optimized for sidelobe attenuation, or pre-identification of neighboring spectral peaks. Nevertheless, for signals dominated by a single fundamental component, the IAE-DFT maintains stable operation, as demonstrated by the presented results.

**Figure 9:**
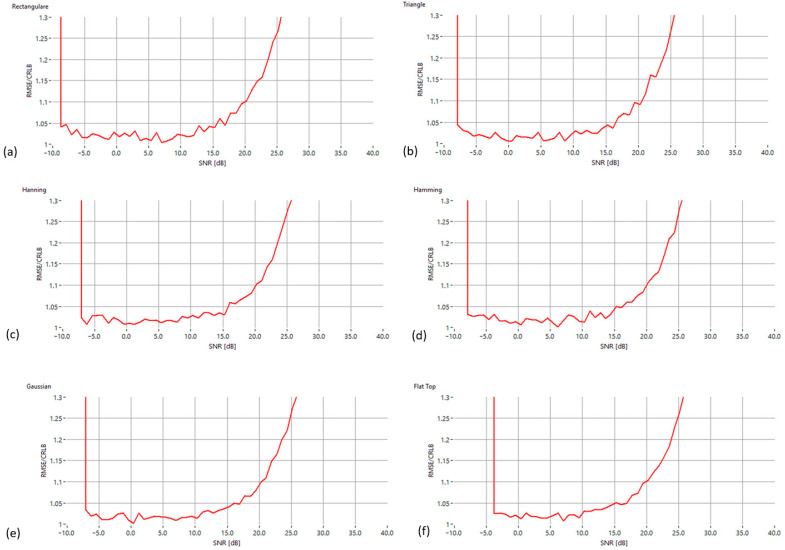
The RMSE/CRLB ratio versus the SNR for six standard window functions applied to the proposed IAE-DFT method: (**a**) Rectangular, (**b**) Triangular, (**c**) Hann, (**d**) Hamming, (**e**) Gaussian and (**f**) Flat-Top.

To evaluate the influence of spectral windowing on estimator performance, the proposed algorithm was tested using six common window functions: rectangular, triangular, Hanning, Hamming, Gaussian and flat-top. Figure 9 displays the RMSE/CRLB ratio versus SNR for each configuration. All windows exhibit a similar behavior: the estimator approaches near-optimal accuracy in the mid-SNR range, with only small deviations from the theoretical bound. Gaussian, Hanning and Hamming windows provide the most stable performance due to their favorable main-lobe symmetry. The rectangular window shows slightly increased sensitivity at very low and high SNR levels, as expected from its higher sidelobe content. The flat-top window presents the largest error in the high-SNR region, consistent with its design focus on amplitude accuracy rather than frequency resolution. Overall, these results confirm that the proposed IAE-DFT algorithm remains robust across different window functions, with improved performance when using windows that preserve strong local spectral symmetry around the dominant peak.

From a qualitative perspective, IpDFT remains appealing for its analytical simplicity, but its accuracy degrades significantly when the true frequency lies close to an FFT bin center. Fractional methods such as FracIpDFT and the hybrid Fan estimator improve performance by evaluating DTFT samples at fractional offsets, which enhances precision at moderate SNR levels but preserves some sensitivity to spectral symmetry points. GFIpDFT provides excellent accuracy at low and medium SNR due to its generalized three-point model, though a small deterministic bias may appear at very high SNR where the main lobe becomes extremely narrow. In contrast, the proposed IAE-DFT replaces analytical interpolation with an iterative symmetry-seeking procedure, which demonstrates stronger noise tolerance at 0 dB and consistent accuracy at higher SNR levels. Together with the quantitative RMSE/CRLB results in Table 1, this qualitative comparison clarifies the contexts in which each method is most suitable and highlights the relative performance of the proposed estimator.

## 5. Conclusions

This paper presented a new frequency-domain approach, IAE-DFT (Iterative Amplitude Equalization in the Frequency Domain), for the accurate frequency estimation of sinusoidal signals. The method relies on a simple iterative process that equalizes the amplitudes of two dominant spectral components by shifting both frequency points in the same direction according to amplitude dominance. Through successive step halving, the algorithm converges deterministically toward the equilibrium region corresponding to the true signal frequency, without requiring phase information or analytical correction formulas.

Simulation results have shown that the proposed IAE-DFT method achieves superior performance under low signal-to-noise conditions (0 dB) and maintains comparable accuracy at higher SNR levels (20 dB and 40 dB). It provides stable and repeatable results even near FFT bin centers, where classical interpolation-based estimators typically degrade. The method’s deterministic convergence make it highly suitable for embedded systems, portable measurement instruments and frequency-output biosensors, where real-time operation and precision are equally important.

Future research will focus on improving the algorithm’s efficiency through adaptive iteration control, including the optimized selection of the initial step size and dynamic adjustment of the step-reduction rate. These refinements are expected to further reduce the number of iterations required and enhance convergence speed, paving the way for practical implementation in high-precision measurement and biosensing applications.

## Data Availability

The data supporting this study’s findings are available from the corresponding author upon reasonable request.

## Appendix A

Input: x[n] -sampled signal fs -sampling frequency N -FFT length max_iter -maximum number of iterations Δf = fs/N -frequency resolution Procedure: 1. Compute FFT of x[n]. 2. Identify two adjacent frequencies with the largest amplitudes: M1 = frequency of dominant bin on the left M2 = frequency of dominant bin on the right 3. Initialize: step = 0.4 * Δf iter = 0 A1 = |FFT amplitude at M1| A2 = |FFT amplitude at M2| prev_sign = sign(A2 − A1) 4. Repeat while iter < max_iter: iter = iter + 1 if A1 > A2: # higher amplitude on the left → move both left M1 = M1 − step M2 = M2 − step else: # higher amplitude on the right → move both right M1 = M1 + step M2 = M2 + step # Recalculate amplitudes using single-point DFT A1 = |DFT(x[n], M1)| A2 = |DFT(x[n], M2)| current_sign = sign(A2 − A1) if current_sign ≠ prev_sign: step = step/2 prev_sign = current_sign 5. Estimated frequency: f_est = (M1 + M2)/2 Output: f_est, iter
